# Effect of spraying of fine water particles on facial skin moisture and viscoelasticity in adult women

**DOI:** 10.1111/srt.12648

**Published:** 2018-11-06

**Authors:** Naoki Nishimura, Shinsuke Inoue, Keiko Yokoyama, Satoshi Iwase

**Affiliations:** ^1^ Faculty of Sport Sciences Nihon Fukushi University Chita Japan; ^2^ Department of Physiology School of Medicine Aichi Medical University Nagakute Aichi Japan; ^3^ Department of L&E Advanced Development Aisin Seiki Co., Ltd. Kariya Japan

**Keywords:** adult women, fine water particles, moisture, skin conductance

## Abstract

**Background/purpose:**

It is known that the elderly and adult women with reduction in sebum secretion have reduced skin barrier function, drying of the skin in a low humidity environment is accompanied by physiological distress. As our hypothesis, when fine water particles are sprayed on the skin, the water content of the corneal layer is significantly increased. In the present study, we examined the ability of fine water particles to improve facial skin moisture levels in adult women.

**Methods:**

We examined skin conductance, transepidermal water loss (TEWL), and skin elasticity as an index of skin barrier function at the cheek in 17 healthy adult women in the spraying of fine water particles, in the environment temperature at 24°C and 34.5% relative humidity.

**Results:**

The skin conductance of stratum corneum after 120 minute of spraying, A condition (peak particle size below 0.5 μm) was 119.7 ± 25.1%, B condition (peak particle size 1.8 μm) was 100.4 ± 31.7%, C condition (peak particle size 5.4 μm) was 110.1 ± 25.0%, and the A condition was significantly higher than the B condition. Also, skin elasticity in the A condition tended to be higher value than in the other conditions. Transepidermal water loss (TEWL) after 120 minute of spraying showed a lower value in the A condition than in the other conditions. In the A condition, the skin conductance steadily maintained their initial levels up to 360 minute after spraying.

**Conclusion:**

Especially, by spraying smallest fine water particles, skin barrier function at the cheek was improved. These data indicated that non‐charged fine water particles played an important role on moisten skin in a low humidity environment.

## 1 PURPOSE

Indoor humidity decreases during heating and cooling in well‐insulated indoor environments and increases water loss from the skin, readily causing dry skin.^1^, ^2^, ^3^, ^4^ In atopic dermatitis patients and the elderly with a reduced skin barrier function, drying of the skin in a low humidity environment is accompanied by physiological and psychological distress. To prevent dry skin, steam, and mist humidifiers are used, but the diameter of water particles formed by many of these devices is in the micron order, and their long‐time use excessively increases indoor humidity and causes condensation, being problematic, such as promoting mold growth.^5^, ^6^, ^7^ We previously demonstrated that fine water particle successfully maintained biophysical parameters such as skin conductance and transepidermal water loss (TEWL) of the face including the lateral canthus in adult women.^5^, ^6^, ^7^

Recently, the development of a humidifier using a conductive polymer material (PEDOT/PSS) has been carried out. The characteristics of PEDOT/PSS are considered as follows: (a) Fine water particles can be released without water supply, (b) since the water particle diameter is 0.5 μm or smaller with non‐charged, released water particles pass through the gap between skin keratinocytes and sebaceous film, permeate the stratum corneum, and increase the water content of the corneal layer, (c) indoor condensation and mold growth are inhibited, reducing discomfort. It is also reported that epidermal hydration significantly increased biomechanical parameters, such as skin distensibility, under suction.^8^

As our hypothesis, when fine water particles formed by a PEDOT/PSS‐equipped device are sprayed on the skin, the water content of the corneal layer is significantly increased compared with the use of steam and mist humidifiers. In this study, water particles with different diameters were sprayed on the facial skin, and the effect on the water content of the corneal layer was investigated. Our data indicated a significant effect of the generated fine water particles on hydration and skin softening.

## 2 METHODS

### 2.1 Subjects

The subjects in our study were 17 healthy adult females aged 30‐46 (mean ± SD: 40.1 ± 5.3 years). The subjects were not required to avoid the period of menstruation when selecting an appropriate experiment day. All experiments were carried out in February. The time of day measurements was taken; the measuring instruments and the measuring personnel were identical for the study.

They were given a sufficient explanation of the study and provided written informed consent. The protocol of the study was approved by the institutional review board of Aichi Medical University. The study was conducted in accordance with the principles of the Declaration of Helsinki.

### 2.2 Characteristics of Spray equipment

Three test conditions were employed with different size of fine water particle, and the measurements for each condition were obtained different days. The peak fine water particle size of each condition was below 0.5 μm in A condition, 1.8 μm in B condition, and 5.4 μm in C condition, respectively. The amount of water released by spraying for 30 minute of each condition was 1.9 g in A condition, 105 g in B condition, and 480 g in C condition, respectively. In addition, the fine water particles in the condition A are non‐charged and continuous operation with no water supply is possible. Steaming equipment under C condition was sprayed with fine water particles warmer than the other conditions.

### 2.3 Experimental protocol of moisturizing effect

All subjects prohibit excessive exercise on the day of the experiment and requested to come to the laboratory at least 30 minute before experimental sessions. Biophysical measurements were performed in an artificial climate‐controlled chamber that was adjusted to ambient temperature at 24°C and 34.5% relative humidity. After wearing the top and bottom of the trainer, the subjects cleansed their face using a cleansing cream. After a 60 minute sitting position in an artificial climate‐controlled chamber, fine water particles of either condition of A to C were sprayed on the left half side of the face for 30 minute. In B and C conditions, spraying of fine water particles was continued for 30 minute, but intermittently sprayed for every 2 minute in A condition. After completion of fine water particle application, subjects recovered 120 minute in the same posture as at rest period. Skin conductance (SKICON 200‐EX^**®**^, IBS Co., Ltd., Hamamatsu, Japan), TEWL (Vapometer^**®**^, Delfin Technologies Ltd., Kupio, Finland) and skin distention (Cutometer^**®**^, Courage & Khazaka, Cologne, Germany) at the cheek area were measured every 60 minute.

### 2.4 Experimental protocol of sustainability of moisturizing effect

Four healthy adult females aged from 21 to 42 (mean ± SD: 31.5 ± 12.1 years) participated in the sustainability experiment of moisturizing effect. In this experiment were carried out at least a week after the moisturizing experiment under the same experimental conditions. After a 60 minute sitting position in an artificial climate‐controlled chamber, fine water particles of either condition of A and B were sprayed on the left half side of the face for 30 minute. Spraying the fine moisture particles in A condition was intermittently every 2 minute. After completion of fine water particle application, all parameter of recovery period of 360 minute was obtained.

### 2.5 Parameters

Skin conductance (μS) is an index of stratum corneum (SC) hydration state. The state of SC hydration was determined by measuring the conductance of high‐frequency current at 3.5 MHz using an impedance meter by the SKICON 200‐EX^**®**^. Technical descriptions of the conductance measuring system have been published by Tagami.^9^ From a total of five readings at cheek area, the mean value of three readings after eliminating the highest and lowest values was adopted for the measurements at site.

TEWL (g/m^2 ^h) an index of the skin barrier functionality is the mass of water that evaporates from the skin surface through a small hole in the measuring device. The Vapometer^**®**^ is a closed chamber instrument which measures TEWL. It is regarded as the most suitable noninvasive method to examine the barrier function. The mean value of two measurements taken at two adjacent areas was adopted for the measurements at each site.

Skin elasticity was analyzed by means of the Cutometer^®^, which is a noninvasive and quantitative method. The skin suction equipment had a measuring probe with a hole of 2 mm in diameter, and time strain mode was employed at negative pressure of −350 mbar load for 2 seconds (on‐time) followed by a relaxation time of 2 seconds (off‐time). For analysis of the elasticity of the skin, R7 was considered. R7 = Ur/Uf: “Portion of the elasticity compared to the complete curve.” Ur (mm) was measured as the immediate relaxation when the suction was switched off. Ur depends mainly on elasticity. Uf was immediately measured as the total skin distention including the elasticity‐dependent part, and Ur/Uf is used to estimate elasticity. R7 are expressed in millimeters as the mean of three readings from the cheek area.

### 2.6 Statistics

All data were expressed as mean ± SD. Statistical significances between sprayed conditions were calculated using two‐way analysis of variance (ANOVA) followed by Greenhouse‐Geisser or Huynh‐Feldt multiple comparison tests. *P*‐values <0.05 were considered significant.

## 3 RESULTS

### 3.1 Skin conductance as a biophysical parameter

**Figure 1:**
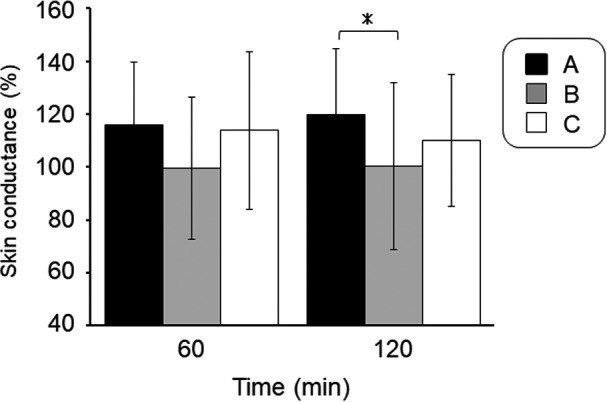
Effect of spraying of fine water particles on the skin conductance of the facial skin. *(*P* < 0.05)

Change in skin conductance of the cheek area after spraying of fine water particles against prior to spraying is shown in Figure 1. The skin conductance of stratum corneum after 60 minute of spraying showed a higher value in the A condition than in the other conditions. Also, 120 minute after the spraying, the A condition was 119.7 ± 25.1%, the B condition was 100.4 ± 31.7%, the C condition was 110.1 ± 25.0%, and the A condition was significantly higher than the B condition (*P* < 0.05).

### 3.2 TEWL as a biophysical parameter

**Figure 2:**
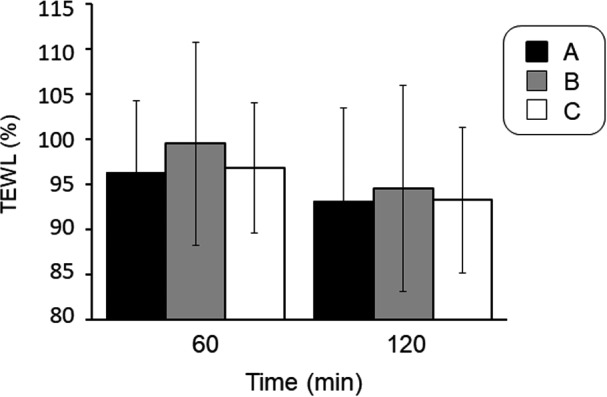
Effect of spraying of fine water particles on the transepidermal water loss (TEWL) of the facial skin

Change in TEWL of the cheek area after spraying of water particles against prior to spraying is shown in Figure 2. Transepidermal water loss showed the lowest value in the A condition, whereas the B condition showed the highest value at both 60 minute and 120 minute after the spraying. However, there were no significant differences between the conditions.

### 3.3 Skin elasticity as a biomechanical parameter

**Figure 3:**
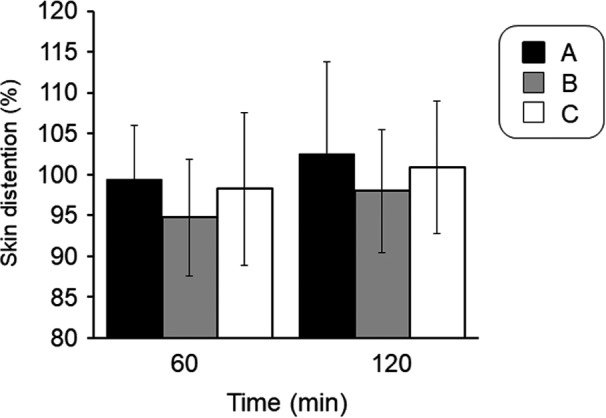
Effect of spraying of fine water particles on the skin distention of the facial skin

Change in skin distention of the cheek area after spraying of water particles against prior to spraying is shown in Figure 3. Skin distention showed the highest value in the A condition at both 60 minute and 120 minute after the spraying. However, there were no significant differences between the conditions.

### 3.4 Sustainability of moisturizing effect by spraying of fine water particles

**Figure 4:**
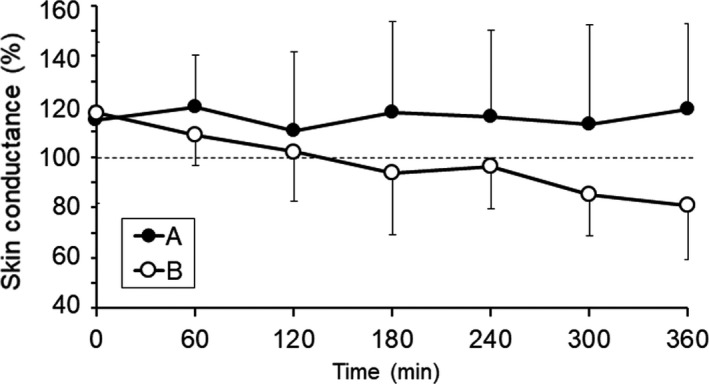
Time course of skin conductance of the facial skin after spraying of fine water particles

Time course of skin conductance of the cheek area up to 360 minute after spraying of fine water particles is shown in Figure 4. In the A condition, the skin conductance steadily maintained their initial levels, while gradually decrease up to 360 minute in the B condition.

## 4 DISCUSSION

This study examined the effects of spraying fine water particles of different diameters on skin conductance, TEWL, and distention of facial skin in adult women.

At 120 minute after spraying fine water particles, skin conductance was increased in comparison with baseline under all conditions (Figure 1). Under all conditions in this study, the diameter of the fine water particles sprayed was smaller than the intercellular spaces of the stratum corneum (approximately 50 nm). Therefore, the sprayed fine water particles may have increased skin conductance by permeating the stratum corneum. However, the degree of increase differed; specifically, the increase was significantly higher in condition A than in condition B. Natural moisturizing factor (NMF) is present in the stratum corneum and consists of filaggrin‐derived low molecular weight amino acids and soft keratin fibers, both of which play important roles in skin barrier function and moisture retention.^10^ To increase skin conductance, the fine water particles must pass through the intercellular spaces and into the stratum corneum. However, NMF is highly hydrophobic, which prevents fine water particles from easily entering the stratum corneum. Here, we surmised that skin conductance was highest in condition A because its water particles had the smallest diameter and were non‐charged, allowing them to permeate the epidermal layer to the dermal layer.

Production of filaggrin, the source of NMF, is known to decrease due to aging and skin dryness.^11^ Reduced filaggrin production results in reduced barrier function. This study was conducted in February, when skin can become particularly dry. Barrier function is considered to depend on the size of the corneocytes and the thickness of the stratum corneum.^10^ Thus, measurements were taken in the stratum corneum of the face, which is thinner than the stratum corneum of the trunk and the extremities and therefore has lower barrier function.^12^ Thickness also differs among different sites on the face. For example, the thickness of the stratum corneum of the cheek, where measurements were taken is 0.015 mm, which is thinner than the stratum corneum in the forehead (0.024 mm). Therefore, spraying fine water particles on the facial skin of adult women is considered useful.

Transepidermal water loss, an indicator of the volume of water loss from the skin, was lowest in condition A (Figure 2). Water loss from the skin, such as through perspiration, emulsifies with sebum to form the sebum membrane, which prevents excessive water loss from the skin and is thus used as an indicator of barrier function. For this reason, TEWL is negatively correlated with skin conductance and sebum volume.^12^ The reduction in sebum secretion in middle‐aged women indicates increased TEWL. In addition, this study was conducted in winter, when transpiration is also decreased. Therefore, TEWL may be increased due to insufficient sebum membrane formation. TEWL was lowest in condition A possibly because the fine water particles formed water molecules on the surface of facial skin and emulsified with sebum to form a sebum membrane, thereby increasing barrier function.

Skin distension was highest in condition A at both 60 and 120 minute after spraying of fine water particles (Figure 3). Human skin undergoes various morphological, physiological, and biochemical changes with age.^10^, ^13^ Beginning around menopause, due to a decrease in estrogen levels, women experience a reduction in skin moisturizing function and distension.^14^, ^15^ Reduced skin moisturizing function is considered to stem from reduced barrier function resulting from a decrease in the intercellular lipid ceramide, while reduced elasticity and distention are considered to result from a reduction in fibroblasts.^10^, ^16^ The reduction in sebum observed in middle‐aged women may also reduce skin barrier function and elasticity. Skin distension is also greatly affected by water volume in and function of not only the epidermal layer, but also the dermal layer. The dermal layer, the inner layer of the epidermis, is composed primarily of collagen produced by fibroblasts. Estrogen contributes to the activity of fibroblasts. The reduction in skin distension among the subjects in this study may have been due to their being in an age range where estrogen secretion is reduced.

Skin conductance in condition A at 360 minute after spraying of fine water particles was at the same level as immediately after spraying (Figure 4). Condition A also demonstrated a significant increase in skin conductance (Figure 1) and the lowest level of TEWL (Figure 2). These results might be attributable to the following: (a) the sprayed fine water particles were dispersed as water molecules on the surface of facial skin, leading to the formation of a sebum membrane, which increased barrier function; and (b) water particles permeated the stratum corneum and collected not only in the epidermal layer, but also in the dermal layer. Corneocytes store as much as approximately 25% by weight bound water,^17^ while the transdermal absorption rate of the stratum corneum increases at temperatures of 32‐39°C.^18^ The fine water particles in this study were sprayed from a device at a warm temperature, which may have improved water retention function.

## 5 CONCLUSION

Spraying fine water particles onto the facial skin of adult women in winter, when skin is dry, improved skin conductance and skin distension. In addition, water retention function remained constant at 360 minute after spraying. The keys to this water retention function are that the diameter of the sprayed fine water particles is smaller than the intercellular spaces and that the particles are non‐charged. Also, condition A is highly convenient because fine water particles can be sprayed repeatedly without replenishing water.
